# First report of *Cladobotryumverticillatum* (Ascomycota, Hypocreaceae) causing cobweb disease on *Paxillusinvolutus*

**DOI:** 10.3897/BDJ.10.e87697

**Published:** 2022-10-11

**Authors:** Xiaoya An, Guohui Cheng, Hanxing Gao, Yang Yang, Dan Li, Changtian Li, Yu Li

**Affiliations:** 1 Engineering Research Center of Chinese Ministry of Education for Edible and Medicinal Fungi, Jilin Agricultural University, Changchun, China Engineering Research Center of Chinese Ministry of Education for Edible and Medicinal Fungi, Jilin Agricultural University Changchun China; 2 College of Plant Protection, Shenyang Agricultural University, Shenyang, China College of Plant Protection, Shenyang Agricultural University Shenyang China; 3 Environment and Plant Protection Institute, Chinese Academy of Tropical Agricultural Sciences, Haikou, China Environment and Plant Protection Institute, Chinese Academy of Tropical Agricultural Sciences Haikou China

**Keywords:** Paxillaceae, Hypocreaceae, mycoparasite, cobweb disease

## Abstract

*Paxillus*, a type of ectomycorrhizal fungi distributed widely in the world, is also an essential category for researching bioactive substances and pharmacological functions. We discovered fruitbodies of *Paxillusinvolutus* covered in a layer of white mycelium in 2020. *Cladobotryumverticillatum*, a pathogenic fungus related to cobweb disease, was isolated and identified based on morphological and phylogenetic features. Koch's postulates were used to confirm the pathogenicity. The host range test revealed that *C.verticillatum* could cause disease in all examined mushrooms except *Ganodermasichuanense*. To our knowledge, *C.verticillatum* is a new record species in China and a new pathogen on *Paxillusinvolutus*.

## Introduction

*Paxillus* Fr. is a genus in the family Paxillaceae, order Boletes. Its members form typical ectomycorrhizal structures with a variety of wooden hosts (Wallander and Söderström 1999) and are distributed throughout the Northern Hemisphere in a variety of ecosystems and habitats (Jargeat et al. 2014). Although it can cause severe anaphylactic reactions when used improperly in cooking, *Paxillusinvolutus* is an important edible mushroom (Dai et al. 2010, Sayyed and Hussain 2020). Recent studies on *Pa.involutus* have focused on the symbiotic mechanism with related trees (Ma et al. 2014) and bioactive substances (Antkowiak et al. 2003, Mikołajczyk and Antkowiak 2009, Lv et al. 2021). Additionaly, *Pa.involutus* has pharmacological functions such as antioxidant, anticancer and antibacterial activities of its metabolites. It can also disperse blood stasis and dehumidification (Kalyoncu et al. 2010, Liu et al. 2018, Zhang et al. 2020). However, there have been few reports of disease on it.

Mycoparasites are an important ecological category that interacts with other fungi (including parasites and saprobes) (Gams et al. 2004, Sun et al. 2019), particularly the genus *Hypomyces*/*Cladobotryum* (Hypocreaceae, Hypocreales), which can cause mushroom cobweb diseases (Põldmaa 2000) and cause significant economic losses for the global edible fungi industry (Bhatt and Singh 2002, Adie et al. 2006). Identification of *Hypomyces*/*Cladobotryum* species depends heavily on the colour of the subicula and perithecia as well as the characteristics of the ascospores (Zeng and Zhuang 2019). Discomycetes (Rogerson and Samuels 1985), Boletales (Rogerson and Samuels 1989), Polyporales (Rogerson and Samuels 1993), and Agaricales (Rogerson and Samuels 1994), are all possible hosts for the genus. Currently, most of the reports about cobweb disease occurred in the cultivation process and focused on artificial edible mushrooms. On the contrary, we paid less attention to cobweb disease in the wild.

In August 2020, we discovered *Paxillusinvolutus* basidiocarps covered with a layer of white mycelium in the Changbai Mountain Biosphere Reserve (CMBR), Jilin Province, China. Broad-leaved forests with *Quercusmongolica* and *Betulaplatyphylla* as the primary tree species supported the diseased fruitbodies. Crippled and decaying mushrooms were collected (42°52′N, 127°81′E). In this paper, we present our findings from natural infestations of *Pa.involutus* fruiting structures with strongly sporulating ascomycetous mycopathogens. We isolated a fungus of *C.verticillatum*, a pathogen of cobweb disease, and investigated its morphology and pathogenic potential. The internal transcribed spacer (ITS), translation elongation factor 1-α (TEF1) and RNA polymerase II subunit (RPB2) were combined and analysed to confirm the identification. We also conducted infection ability tests using the fruiting bodies of other basidiomycetous species.

## Materials and Methods

### Fungal isolation

Diseased fruitbodies were cut into small pieces (5 mm × 5 mm × 5 mm) with a sterilised scalpel, and infected tissues were immersed in 75% ethanol solution for 45 s before being rinsed three times with sterilised water. Then, dried surface with sterile filter paper, placed on Potato Dextrose Agar (PDA) plates containing 100 mg/l streptomycin sulphate, incubated at room temperature, and transferred the culture to fresh PDA plates when the fungal hyphae emerged and cultured the plates at 25°C for five days to allow the colonies to sporulate fully. Use the single spore separation to get the pure cultures following the method described by Chomnunti et al. (2014). The spore suspension was obtained by washing the spores with 10 ml of sterile water into the Petri dishes and diluted to a final concentration of 5×10^2^ conidia/ml using a blood count plate. Then, the prepared spore suspension (100 μl) was placed uniformly on Petri dishes containing a 2- to 3-mm-thick layer of 2% water agar (WA) medium (20 g agar powder, 1000 ml water). After being incubated at 25°C for 12 hours, single colonies were picked on a new PDA plate with a sterile needle by observation under a microscope, thereby obtaining pure colonies. Store the strains at 4°C in the Engineering Research Center of Edible and Medicinal Fungi, Ministry of Education, Jilin Agricultural University (Changchun, Jilin, China).

### Morphology

After activating the pathogen, picked some hyphae with the inoculation needle from the culture and transferred them on a slide aseptically for morphological identification. Mycelial samples with conidiophores and conidia were observed under a Zeiss Axio Lab A1 light microscope (Carl Zeiss, Germany) and microscopic observations made with objectives of 10x, 20x, 40x and 100x oil immersion. All measurements and photographs were performed using a Zeiss Imager A2 microscope with an Axiocam 506 colour camera and integrated software. Microscopically, the characteristics of 30 conidia and conidiophores from the isolates were observed. Morphological identification was performed using the Gams and Hoozemans (1970) and Seifert and Gams (2011) methods.

### DNA extraction and PCR amplification

The genomic DNA of the pathogen (*C.verticillatum*) was extracted from the mycelia of colonies on PDA. Gene sequences of ITS, TEF1 and RPB2 were amplified by a polymerase chain reaction (PCR) with the primer pairs of ITS4/ITS5 (White et al. 1990), EF1-983F/EF1-2218R (Rehner and Buckley 2005) and RPB2-5F/RPB2-7Cr (Liu et al. 1999), respectively. The reaction included an initial denaturation at 95°C for 5 min, followed by 35 cycles of denaturation at 95°C for 60 s, annealing at 55°C for 60 s for RPB2 (incrementally increasing by 2 s), 54°C for 50 s for ITS, 55°C for 60 s for TEF1, extension at 72°C for 60 s and a final extension at 72°C for 10 min, using an Applied Biosystems S1000^TM^ Thermal Cycler. PCR products were sent to the Changchun Branch of Sangon Biotech Co., LTD for sequencing and confirmed by BLAST on NCBI (https://blast.ncbi.nlm.nih.gov/Blast.cgi). The strains and the NCBI Genbank accession numbers of DNA sequences used in this work are listed in Suppl. material 1.

### Phylogenetic analyses

BLASTn searches with the sequences were performed against NCBI to detect the most closely-related species (http://www.blast.ncbi.nlm.nih.gov/). Phylogenetic trees were constructed using ITS, TEF1 and RPB2 sequences, and phylogenetic analyses were performed with the Maximum Likelihood (ML) and Maximum Parsimony (MP) methods. Multiple alignments of all present sequences were automatically generated using MAFFT V. 7.471, and manual improvements were made using BioEdit when necessary (Hall 1999, Katoh and Standley 2013), and converted to nexus and NEX format through the software Aliview (Larsson 2014). In the analysis, ambiguous areas were excluded and gaps were regarded as missing data. The Maximum Parsimony phylogram (Swofford 2003) was constructed with PAUP 4.0a 167 from the combined sequences of ITS, TEF1 and RPB2, using 1000 replicates of heuristic search with random addition of sequences and subsequent tbr (tree bisection and reconnection) branch swapping. Analyses were performed with all characters treated as unordered and unweighted, with gaps treated as missing data. Maximum Parsimony bootstrap proportion (MPBP) was used to test the topological confidence of the resulting sequences with 1000 replications, each with ten replicates of random addition of taxa. An ML phylogram was constructed with raxmlGUI 2.0 (Edler et al. 2020) with the sequence after alignment. The ML+ Rapid bootstrap program and 1000 repeats of the GTRGAMMAI model were used to evaluate the bootstrap proportion (BP) of each branch for constructing the phylogenetic tree.

### Koch's postulates and host range test

The experiments were carried out in duplicate to confirm the pathogenicity of the strain YW, according to Koch's postulates. We found *Pa.involutus* fruiting bodies in a birch forest on the campus of Jilin Agricultural University and inoculated them with spore suspension (50 μl) on caps. We observed the process in the wild and recorded changes in disease symptoms for ten days. Select the fruiting body with white mycelium for fungal isolation. Furthermore, the host range tests were investigated by inoculating it on to nine commercial mushroom species: *Pleurotusostreatus*, *Hypsizygusmarmoreus*, *Agrocybeaegerita*, *Pleurotusgeesteranus*, *Pleurotuscitrinopileatus*, *Flammulinafiliformis*, *Pleurotussalmoneostramineus*, *Ganodermasichuanense* and *Agaricusbisporus*. All mushrooms were grown on the substrate and kept in the growing station. Mushrooms were inoculated with one droplet (50 μl) of spore suspension (5 × 10^6^ unit/ml) mixed with Tween 80 on the upper surface of caps when they reached 3 to 4 cm in diameter (*Pl.ostreatus*, *Pl.salmoneostramineus*, *Pl.geesteranus*, *Pl.citrinopileatus*, *Aga.bisporus*, *Hyps.marmoreus*, *Agr.aegerita*) or stipe (*F.filiformis*). For *G.sichuanense*, inoculated the spore suspension on the solid layer under the pileus. Placed all mushroom bags at 25℃ and kept the air humidity at 80%­–90%. The incident was observed and photographed.

## Results

### Morphological characteristics

**Figure 1.**
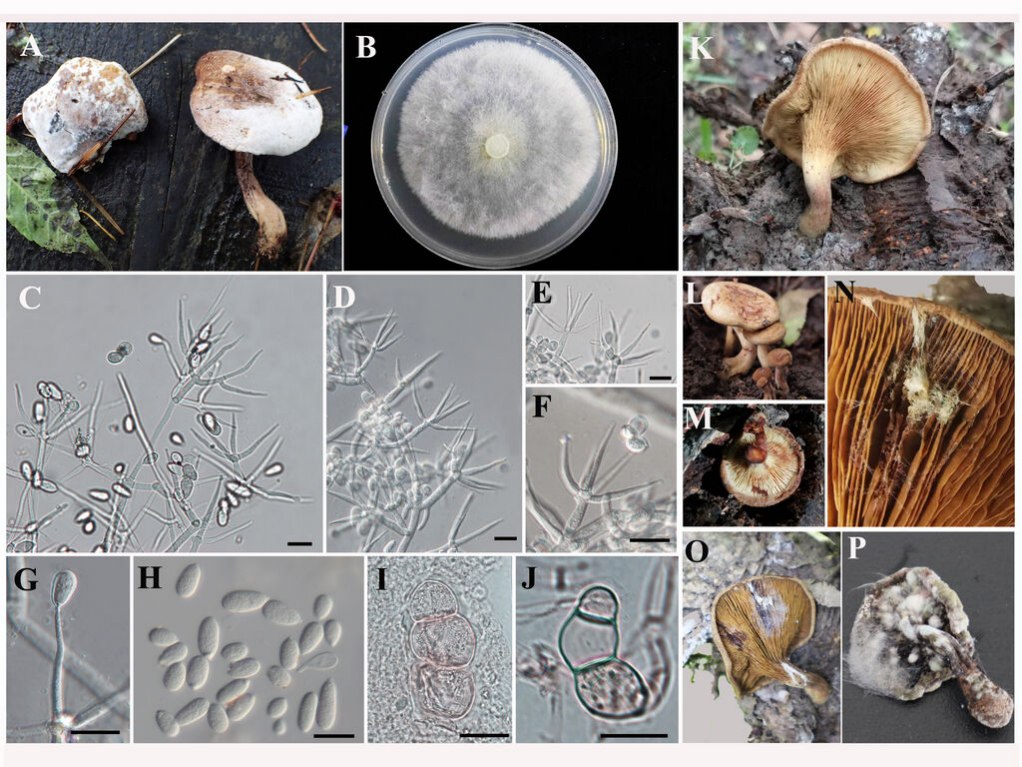
Field symptoms and morphological characteristics of *Cladobotryumverticillatum*. **A** Diseased fruiting bodies in the wild; **B** Colony on PDA; **C** Conidiophores with whorled and single phialides; **D**-**F** Tapered conidiogenous cells form singly or in whorls; **G** Conidiogenous cells; **H** Conidia; **I, J** chlamydospore; **K, L** Healthy mushrooms in the wild; **M-P** Mushrooms artificially inoculated with pathogens at 24 h, 48 h, 72 h and seven days, respectively. Bars: C–G, I, J = 20 μm; H = 10 μm

Colonies spread, appearing fluffy, lanose, tufted or fine linen, white, with suberect tufts about 1–2 cm high, at length sinking and fading. Mycelium is branched, septate and hyaline with rich inclusions. Hyaline conidiophores have one to three septa, are verticillately or irregularly branched and their carriers are branched into two to five phialides. Conidia are 10.6–16.2 × 6.6–11.1 µm, one-celled, smooth- and thin-walled, hyaline, elliptical or elliptical-oblong, with protruding basal scars (Fig. 1). The characteristics agreed with the description of *C.verticillatum* offered by Hoog (1978) and Rogerson and Samuels (1989).

### Phylogenetic analyses

**Figure 2.**
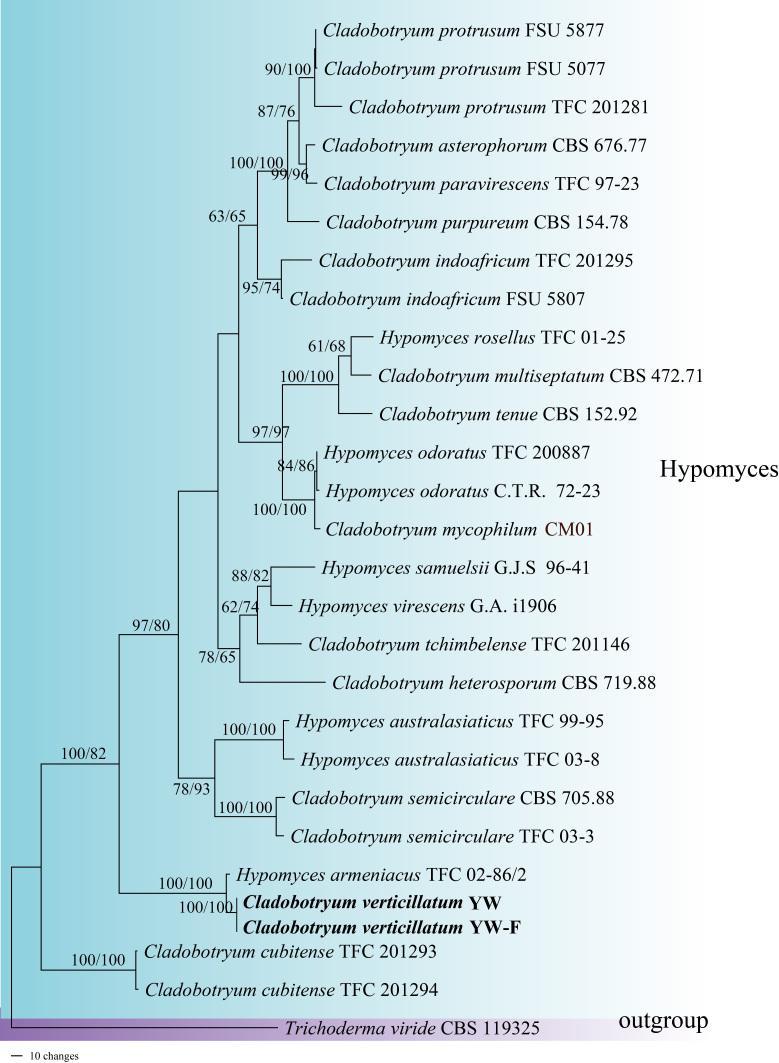
Maximum Parsimony phylogram reconstructed from the combined sequences of ITS, TEF1 and RPB2, showing the species’ phylogenetic position. Bootstraps above 50% (MP left/ML right) are given, respectively. The new sequences are shown in bold.

The BLAST results showed that the ITS sequence of strain YW was 99.83% similar to MT237489, the RPB2 sequence was 99.72% similar to FN868678, and the TEF1 sequence was 99.02% similar to FN868742, respectively. The dataset for phylogenetic analyses contained 27 ITS sequences, representing 19 species, choosing *Trichodermavirid* as the outgroup taxon. Multi-locus data were concatenated, which comprised 2554 characters with ITS 597 characters, TEF1 888 characters and RPB2 1069 characters. Estimated base frequencies were as follows: A = 0.233413, C = 0.296140, G = 0.248170 and T = 0.222278; substitution rates AC = 1.489328, AG = 3.647092, AT = 1.111646, CG = 0.925803, CT = 7.920581 and GT = 1.000000. In the resulting tree (Fig. 2), the combined phylogenetic analyses using ITS, TEF1 and RPB2 showed that our strains were clustered with the sequences of *Hyp.armeniacus* (the teleomorph name of *C.verticillatum*) in a branch with high statistical support (MPBP/MLBP = 100%/100%). The phylogenetic tree indicated that the pathogen was *Hyp.armeniacus*. However, we did not observe any characteristics of the teleomorph phase. Thus we named it *C.verticillatum*. The branch of YW was most related to the clade that contains *C.cubitense* and *C.semicirculare*. The MP and ML trees showed similar topologies with high statistical support values, and the MP tree was selected as the representative phylogeny (Fig. 2). The bootstrap values (BP) ≥ 50% were shown on the branches. The sequences of *Pa.involutus* have been submitted on Genbank with accession numbers ITS-OL659295, TEF1-OP243230 and GPD-OP243231.

### Fruiting body infection tests

The pathogenicity test revealed that all the inoculated *Pa.involutus* exhibited first symptoms after 24 hours, with taupe lesions appearing on the surface of the gills (Fig. 1). White hyphae then appeared and spread through the gills of *Pa.involutus* (Fig. 1). The pileus and stipe surfaces were covered in fluffy white mycelium that resembled spider webs 72 hours after inoculation (Fig. 1). After seven days of incubating at 25°C, pathogenic mycelium formed white spots on the surface of fruiting body, and the casing shrivelled and wilted (Fig. 1). Ten days later, the gills had decayed and turned black, brown water droplets had exuded from the collapsed fruiting bodies, and the pathogen's white hypha had vanished. During this process, we were able to re-isolate and identify the pathogen from the infected fruiting bodies and obtained the strain YW-F, which was stored at Jilin Agricultural University. This species was identified as the same as YW, and the ITS, RPB2 and TEF1 sequences have been submitted on Genbank. The accession numbers are shown in Suppl. material 1.

**Figure 3.**
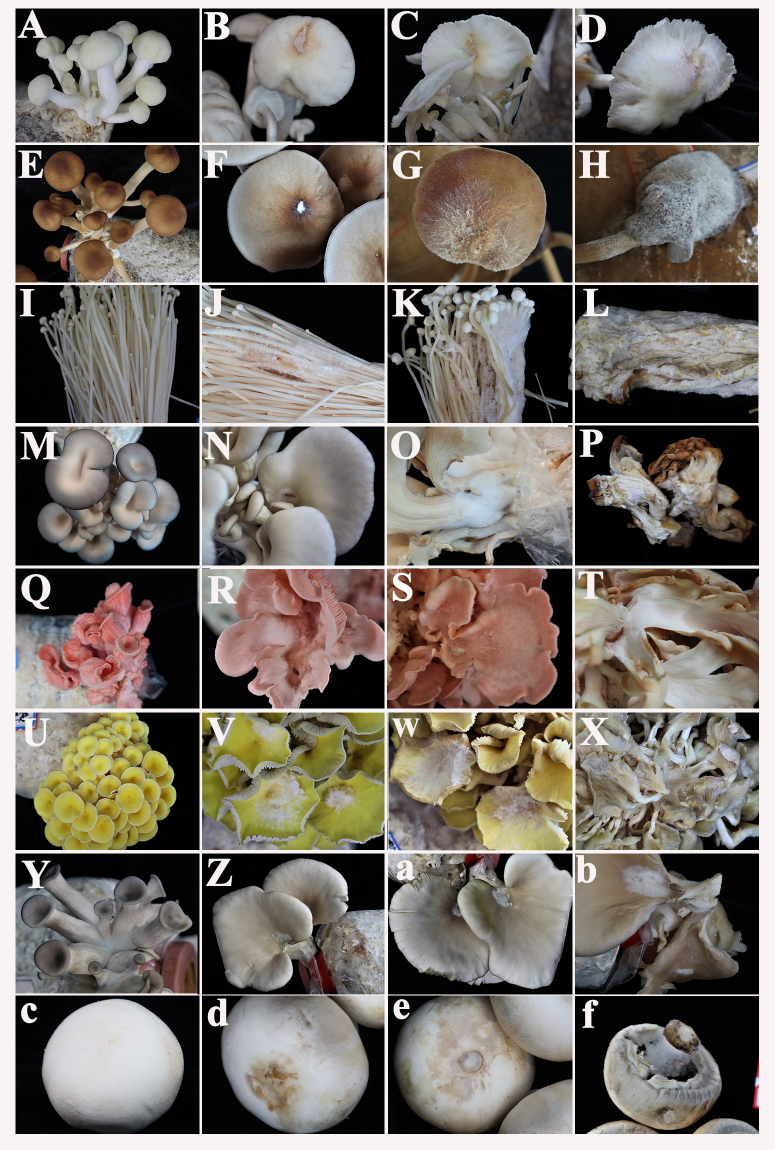
Disease development on different mushrooms after inoculating *Cladobotryumverticillatum*. **A–D** Pictures of *Hypsizygusmarmoreus* in healthy condition, 1 dpi, 2 dpi and 3 dpi; **E–H** Pictures of *Agrocybeaegerita* in health, 1 dpi, 2 dpi and 3 dpi; **I–L** Pictures of *Flammulinafiliformis* in health, 1 dpi, 2 dpi and 4 dpi; **M–P** Pictures of *Pleurotusostreatus* in health, 1 dpi, 2 dpi and 8 dpi; **Q–T** Pictures of *Pleurotussalmoneostramineus* in health, 1 dpi, 2 dpi and 7 dpi; **U–X** Pictures of *Pleurotuscitrinopileatus* in health, 1 dpi, 2 dpi and 3 dpi; **Y–b** Pictures of *Pleurotusgeesteranus* in health, 1 dpi, 2 dpi and 3 dpi; **C–f** Pictures of *Agaricusbisporus* in health, 1 dpi, 2 dpi and 3 dpi.

The strain YW was tested on nine commercial mushroom types and found to be capable of infecting all but *G.sichuanese*. After inoculating the spore suspension on the stipes or caps of fruiting bodies, the hyphae began to grow. Typical cobweb signs, such as small brown spots, were seen 1–3 days post-inoculation (dpi). The white mycelia were then visible, and the fruiting bodies were rotting and covered in massive spores after 3-5 days. Finally, the mushrooms wilted and rotted, mirroring the characteristics of the field sample (Fig. 1). However, the extent and duration of wilting of edible fungi varied due to host differences (Fig. 3).

Disease processions on *F.filiformis*, *Aga.bisporus*, *Agr.aegerita*, *Hyps.marmoreus* and *Pl.citrinopileatus*, were usually completed within four days and caused serious damage. White hyphae were visible on the first day post-inoculation and spread quickly, causing the mushrooms to become soft and brown. Symptoms of *Aga.bisporus* and *Hyp.marmoreus* were similar, with brown spots and mycelia visible at the inoculation site. Mycelia eventually covered the cap of *Aga.bisporus* and spread to the stalk-cap junction, resembling a spider's web. *Pleurotuscitrinopileatus*, unlike others, had no brown spots on the cap, but as the hardness decreased or even disappeared, it eventually fell in clusters and turned brown.

Although *Pl.ostreatus*, *Pl.salmoneostramineus* and *Pl.geesteranus* displayed symptoms earlier, the progression was slow and prolonged. The hyphae grew on the first day, but no lesions were visible. Hyphae continued to stretch, causing the mushrooms to stop growing and atrophy, and the surface of the fruitbodies to become covered in white mycelia. When inoculated on the primordium or cap, *G.sichuanense* showed less sensitivity or high resistance to the pathogen when compared to other edible fungi.

## Discussion

Based on morphological and molecular characteristics, we isolated a fungal pathogen from diseased *Pa.involutus* and identified it as *C.verticillatum*. It was originally described by Heinrich and named by Hughes (1958). Amongst members of the Hypocreales parasitising agaricomycetes from temperate to tropical latitudes (Põldmaa 2000, Põldmaa and Samuels 2004), *C.verticillatum* is often found in temperate regions, Colombia, Europe (England, France, Germany, Sweden), Canada and the United States (Rogerson and Samuels 1994), but has never been reported in China before, nor on *Pa.involutus*.

*Hypomyces*/*Cladobotryum* species that live on Polyporales may have a lower host-selection than others (Tamm and Põldmaa 2013). *Cladobotryumarnoldii* (*Hyp.lithuanicus*) and *Hypomyceshyalinus*, for example, were strictly host-specific, living on *Lactariustorminosus* and *Amanita*, respectively. Although *C.verticillatum* occurs mostly on *Russula*, *Lactarius* and *Agaricus* (Hughes 1958, Gams and Hoozemans 1970, Põldmaa 2011), it does not have such strong host specificity or can even grow on the substratum of the actual host when the hosts are destroyed (Rogerson and Samuels 1993, Põldmaa and Samuels 1999). The host range test in this study, however, revealed that it could not cause disease in *G.sichuanense*. McKay et al. (1999) demonstrated that the anamorphs of temperate, red perithecial *Hypomyces* are the causative agents of cobweb disease, which cause epidemics in mushroom farms. *Cladobotryumverticillatum* can also cause cobweb disease on *Aga.bisporus*, causing a botryte-like disease (verticillium wilt) in edible fungi (Fletcher and Gaze 2007). Unlike the common pathogenic species of cobweb disease, *C.mycophilum*, *C.dendroides* and *C.protrusum*, *C.verticillatum* occasionally produces sclerotium, and the mycelia dissolve with increased time on PDA. Furthermore, *C.verticillatum* does not produce pink or red pigments throughout the course of infection, remaining white or light ochre yellow. A large number of conidia can only be observed in the later stage of infection. However, the pathogens for cobweb disease are generally highly adaptable to a wide range of pH (Grogan and Gaze 2000, Adie 2001, Zhang et al. 2015). Therefore, they can better adapt to a variety of habitats and hosts, thus occupying a higher ecological niche.

## Supplementary Material

0865F511-7F0A-53C7-9F32-A81369130221 10.3897/BDJ.10.e87697.suppl1 Supplementary material 1 Strains and specimens of *Cladobotryum*/*Hypomyces* included in the phylogenetic analyses Data type GenBank accession numbers Brief description Accession numbers include details such as locality, isolate numbers of the sequences used for this study File: oo_734628.docx https://binary.pensoft.net/file/734628 Xiaoya An
