# Supplementary material for: First report of Cladobotryumverticillatum (Ascomycota, Hypocreaceae) causing cobweb disease on Paxillusinvolutus
Source: Biodivers Data J. 2022 Oct 11;10:e87697. doi: 10.3897/BDJ.10.e87697 (PMC9836559; doi:10.3897/BDJ.10.e87697)
Supplement: Supplementary material 1 — Strains and specimens of Cladobotryum/Hypomyces included in the phylogenetic analyses [file bdj-10-e87697-s001.docx]

**Table 1:** Strains and specimens of *Cladobotryum*/*Hypomyces* included in the phylogenetic analyses

| **Species** | **Isolate or specimen number** | **Country of origin** | **GenBank accession numbers** | | |
| --- | --- | --- | --- | --- | --- |
|  |  |  | **RPB2** | **TEF** | **ITS** |
| *Cladobotryum asterophorum* | CBS 676.77 | Japan | FN868649 | FN868712 | FN859395 |
| *C. cubitense* | TFC 201293 | Madagascar | FN868676 | FN868740 | FN859422 |
|  | TFC 201294 | Madagascar | FN868677 | FN868741 | FN859423 |
| *C. heterosporum* | CBS 719.88 | Cuba | FN868653 | FN868716 | FN859398 |
| *C. indoafrum* | TFC 201295 | Madagascar | FN868657 | FN868721 | FN859403 |
|  | FSU 5807 | South Africa | FN868654 | FN868717 | FN859399 |
| *C. multiseptatum* | CBS 472.71 | New Zealand | FN868659 | HF911724 | MH860221 |
| ***C. mycophilum*** | **CM01** | **China** | **OL613892** | **OL613894** | **OL588220** |
| *C. paravirescens* | TFC 97-23 | Thailand | FN868660 | FN868724 | FN859406 |
| *C. protrusum* | TFC 201281 | Madagascar | FN868667 | FN86873 | FN859413 |
|  | FSU 5077 | China | FN868664 | FN868728 | FN859410 |
|  | FSU 5877 | Republic of South Africa | FN868665 | FN868729 | FN859411 |
| *C. purpureum* | CBS 154.78 | USA | FN868669 | FN868733 | FN859415 |
| *C. semicirculare* | CBS 705.88^T^ | Cuba | FN868671 | FN868735 | FN859417 |
|  | TFC 03-3 | Sri Lanka | FN868672 | FN868736 | FN859418 |
| *C. tchimbelense* | TFC 201146 | Gabon | FN868673 | FN868737 | FN859419 |
| *C. tenue* | CBS 152.92 | Germany | FN868674 | FN868738 | FN859420 |
| ***C. verticillatum*** | **YW** | **China** | **OL613893** | **OL613895** | **OL588199** |
|  | **YW-F** | **China** | **OP185387** | **OP185386** | **OP179782** |
| *Hypomyces armeniacus* | TFC0286/2 | France | FN868678 | FN868742 | FN859424 |
| *H. australasiaticus* | TFC 99-95 | Australia | FN868680 | FN868745 | FN859427 |
|  | TFC 03-8^T^ | Sri Lanka | FN868681 | FN868746 | FN859428 |
| *H. odoratus* | TFC 200887 | Estonia | FN868693 | FN868757 | FN859439 |
|  | C.T.R. 72-23 | USA | FN868687 | FN868752 | FN859433 |
| *H. rosellus* | TFC 01-25 | France | FN868696 | FN868760 | FN859442 |
| *H.s samuelsii* | G.J.S.96-41 | Puerto Rico | FN868702 | FN868766 | FN859448 |
| *H. virescens* | G.A. i1906 | Cuba | FN868708 | FN868772 | FN859454 |
| *Trichoderma viride* | CBS119325 | South America | EU248599 | AY376053 | AY380908 |

Note: The sequences in bold format are those in this study.
